# The Central N. Y. Hom. Med. Society

**Published:** 1882-12

**Authors:** C. P. Jennings

**Affiliations:** Secretary


### THE CENTRAL NEW YORK HOMŒOPATHIC MEDICAL SOCIETY.

The Central New York Homœopathic Medical Society met in Syracuse, N. Y., the 21st of September, 1882, C. W. Boyce, M. D., in the chair.

Dr. Hawley presented the report of the Committee appointed at the last meeting on consultation with non-homœopathists. Adopted.

Your Committee find that the Code of Ethics of the American Institute of Homœopathy, adopted by this Society, contains all that can be desired on this subject, giving, as it does, to every member of the profession, without dispute, the right to his individual opinion, and to advise with any duly qualified physician, demanding only a legal status in the profession and such courteous treatment of each other as one gentleman would always give another.

They further find that "The New York State Medical Society" has recently placed its Code on the same basis of freedom and gentlemanly courtesy; and that out of this action of that meeting has grown up a discussion among the members of the old school, which has become so general as to attract public attention and is producing in some degree the impression that the homœopathic profession has sought, and is still seeking, such a change in the Code of the old school. They find also, as a further basis of this impression, that there is a somewhat numerous class of *eclectics*, who, having crept into the various homœopathic societies, are so clamorous for recognition by the old school that they are willing to drop our distinctive name, as they have already abandoned every principle of our school, whereby they bring upon the whole homœopathic fraternity the charge of professing one thing and practicing another.

Your Committee further find that the sole difference between the old school and the homœopathic profession is in the administration of *drugs* for the cure of the sick, the latter claiming to have the law of the curative relation of drugs to diseases, while the old school deny that the law is known.

Your Committee therefore offer and advise the adoption of the following preamble and resolution as expressing the sentiments of this Society:

WHEREAS, It is evident that the curative relation of drugs to diseases, if there is such relation, must, like all things else, be governed by law, and the *homœopathic profession has a knowledge of and is guided by that law,* which law has no relation to pathology, diagnostics, prognostics, or practical surgery; therefore,

*Resolved,* That homœopathic physicians, abiding by their Code of Ethics, and giving gentlemanly courtesy to all legally qualified members of the medical profession, *in the administration of drugs* for the cure of the sick, neither *need* nor *ask* counsel of any who, ignorant of the law, are guided only by *empirics,* or, as they phrase it, "the accumulated experience of the profession."

Papers contributed by E. Carleton, M. D, and C. Lippe, M. D., of New York city, were read, and the thanks of the Society were given to those gentlemen.

Dr. Clausen read a paper of clinical cases. Accepted with thanks.

Dr. Nash: I call attention to an article in the *Medical Counselor,* May 31st, 1882, on Ptelia trifoliata, by Dr. J. Preston. It is clinical. Study of the provings and use of the remedy have convinced me that it is a valuable medicine.

Dr. Hawley: A good many provers take massive doses and repeat too soon. Such provings are of doubtful value.

Dr. Boyce: Paragraph 128 of the *Organon* is to the point. Dr. A. E. Wallace gave some time ago a proving of Baptisia. His characteristic symptoms—numbness of left arm, loss of sleep, almost loss of consciousness—were valuable, and have been depended on ever since. We have been many years a society. We have never proved any drug, save Glonoine, which was proved at one time while we were in session. Who of us have the energy to take up a drug and prove it according to this paragraph? The Secretary might select a drug and send a portion to each member to be proved according to this paragraph, and the results could be reported to the December meeting—the drug to be in the 30th potency, and not to be made known till the December meeting.

Dr. Nash: I proposed trial to a physician who has no faith in potencies. He refused.

Dr. Hawley: I cannot remember the time when I was not dyspeptic; and, therefore, have not undertaken to prove drugs. Have no doubt many would obtain effects from the 30th. A patient told me the name of a remedy given to her in the 30th attenuation. I asked her how she knew. She answered that she tested it. She had no other means of knowing.

Nearly all the gentlemen present agreed to prove a drug.

Dr. Nash: Persons may take the 30th for a long time and get no effect from it if no attention be given to diet and to abstinence from tobacco and from stimulants.

Dr. Hawley: I have a case which will interest you. A lady, æt. 66, had what was pronounced by an eminent physician of this city congestion of the spinal cord, there being a throbbing under the shoulder-blade, to the left of the spine. She had taken Morphine for weeks. I was called to her two weeks ago. Put her on Nux vom.$^{30}$, dose every four hours. A new bottle of Morphine had just been bought, and she thought she could not dispense with it. I allowed her to take the Morphine at the same time for six days, stipulating that the bottle should be refilled with water after every dose of Morphine. She had been taking strong cathartics to remove the constipation brought on by the Morphine. A day or two ago she had the diarrhœa and pain and pressure in the rectum, which are characteristic of Nux vom. Were these symptoms produced by Nux vom.? As for any congestion of the spinal cord, who ever heard of it without more or less paralysis being present? There has been no paralysis. I diagnosed hyperæsthesia of the stomach. This made her sensitive to every pulsation of the descending aorta.

Dr. Boyce: I propose that we turn our attention to the materia medica. Let a drug be agreed upon; then let each man tell—not what he has read and heard about it—but what he himself has done with it.

Dr. Hawley suggested Cina, and it was accepted.

Dr. Wallace: Have relieved with Cina a child having these symptoms: high fever, paleness about the mouth and nose, gritting of the teeth in sleep, muscular twitchings in arms and legs.

Dr. Nash: Have done the same thing with Cina. In one case an additional symptom was the milky urine. The 3d and 6th failed. Santonine, in quarter-grain doses, failed. Gave them two weeks' trial, and all in vain. The child's rubbing of the nose pointed to Cina. The 200th gave prompt relief.

Dr. Clausen: Have verified an important symptom pointed out to me by Dr. Hawley, to wit: a slight rheumatic pain in the lower extremities. A child, two years old, not willing to be touched or looked at, fearing increase of pain. Cina gave considerable relief.

Dr. Hawley: Have always seen the symptoms named by Dr. Wallace removed by Cina. Another symptom is constant swallowing, as if the child were sucking candy; swelling and heat of the ankles; the child afraid to have you touch the bed; these yield to Cina when attended by the characteristic symptoms of Cina mentioned by Dr. Wallace. When a child has pains in the joints here and there it is an indication of Cina. Was called to a child last night, the mother supposing it to have diphtheria. The exudation was not superimposed upon the mucous membrane, but was seated in it, on a general level with it, and was a sort of dead white—not glistening—the least bit dirty. Breath fœtid. The child had the characteristic symptoms of Cina spoken of here. It is doing well upon Cina. This experience of rheumatic pains being relieved by Cina has been confined, in my observation, to children. The general condition corresponded to Cina. Can recall many cases in which I have bothered with such rheumatic pains in children which could have been met by Cina if I had known it. Found the symptom in the old *Symptomatum Codex.* With the rheumatic pains dread of motion is prominent.

Dr. Boyce: Have often given Cina when it seemed to be indicated, but Cina failed to wipe the thing out. Dr. Guernsey gives a symptom as having been met by Cina: the child exceedingly cross, fretful, irritable.

Dr. Nash: A young lady, æt. 15, was troubled in her eyesight while studying. It was as if a veil hung before her eyes; removed momentarily by wiping with the hand. Cina$^{200}$ cured her promptly. In an epidemic of typhoid fever a child sickened with fever and was brought very low. It noticed nothing, except when you touched her lips to give her water. A gagging motion led me to give Cina. Gave the 200th. The child recovered. There seems to be in certain children a condition which complicates other symptoms, and which condition is removed by Cina; then indicated remedies will act which before were inert—*i. e.*, it may be so. Severe hacking cough, with looseness of the bowels, is often removed by Cina. My own child seemed to have bronchitis. Belladonna did no good. A

choking cough and gagging led me to give Cina. It cured the case promptly.

Dr. Scudder: Am here as a visitor. Let me say that Dr. Guernsey classes Cina among the anti-psorics.

Dr. Boyce: I suggest conversation upon Tartar emetic. [*Consent.*] I have almost always removed severe colic by Tartar emetic. Nausea and diarrhœa may be present, or may not be present, but the griping pain is relieved by it. Have given the first decimal trituration in water. Have given it till it produced its characteristic effects. I used Tartar emetic at first with the idea of producing nausea, but before that point was reached the colic was relieved. This began to be my experience with Tartar emetic, when, as yet, I knew nothing of Homœopathy, and Tartar emetic is a strong remedy in the homœopathic treatment of colic. In croup, also, I have used it successfully when the face was blue.

Dr. Nash: To give Tartar emetic in colic, in the first decimal, till nausea is produced—is that Homœopathy? If you produce nausea you relax spasm. When Tartar emetic is thoroughly homœopathic to a case of colic it will not be necessary to give low attenuations nor to produce nausea.

Dr. Boyce: We are telling, now, what we have done with Tartar emetic, and I am not afraid to tell what I have done. It is Homœopathy. The colic is the Colocynth variety.

Dr. Nash: A doctor told me he had a specific for colic, viz., ten drops of Tincture of Lobelia!

I have cured severe cases of cholera morbus with Tart. em. dynamized.

Dr. Seward: Colocynth has pinching in the bowels and cramping in the legs.

Dr. Wallace: Cured a skin disease in a boy of twelve; the pustules came in crops, and resembled small-pox pustules, except that they lacked the depression in the centre. Cured with one dose of the sixth of Tart. em. I always think of Tart. em. in cholera morbus. A lady had been treated allopathically. Found intense nausea and vomiting; could not keep down cold water; Tart. em.$^{3}$ relieved her. After a few doses an eruption of pustules came on. The pustules dried down and peeled off, as small-pox pustules do. An eruption resembling that which allopathists produce with Tartar emetic ointment always suggests to me Tart. em.

Dr. Hawley: Did a curious thing once with Tart. em. A child three to five months old had congenital syphilis, such as the old school consider necessarily fatal. The sores were all over the child. He could not bear to be touched or approached. Tart. em. cured him right along. Now, at four or five years of age, he is bright and healthy. Have often removed a loose, rattling cough with Tart. em.

Dr. Nash: Have cured the characteristic Tart. em. cough in children and in very old people with Tart. em. Also, cholera morbus where there were these symptoms: great effort in vomiting, great chilliness, and great sleepiness. Tart. em., Opium, and Nux moschata have heavy sleepiness. That of Tart. em. has paleness of face, that of opium a red face and stertor. Have cured intermittent fever with Tart. em. when the patient slept continually during the sweat and heat and was prostrated.

Dr. Brewster: Had a case of typhoid pneumonia, a boy. He had been ten days under allopathic treatment. Council had been held. A priest had been called to perform the last rites of his Church. I found him in a stupor; profuse perspiration; rattling in the chest; difficulty of breathing; spitting almost constantly a watery slime, partly bloody. Patient could not lie down. Tart. em.$^{3}$ in water, a teaspoonful every two hours. Six days afterward he walked to my office a well boy. Had a case of croup, a recent attack. Mucous lining inflamed and pushed up so as to threaten suffocation; stupor; thirst. Tart. em. relieved promptly. I often give it in the second stage of croup.

Dr. Seward: Have had a similar experience. Also Tart. em. cured for me a case of pimples, surface covered.

Dr. Gwynn: In croup I am guided to Tart. em. by a peculiar redness of the papillæ of the tongue and rattling in the bronchi. In pneumonia, when the child wants to be carried upright in the arms, Tart. em. has cured. Also, with cold sweat, the sweat being colder than the patient. In the Veratrum album sweat the patient is icy cold, like a corpse.

Dr. Jennings: Have seen Tart. em. cure pneumonia where the child must be carried upright in the arms.

Dr. Besemer: Tart. em. relieves advanced cases of loaded bronchi where there is loose, rattling cough, and it seems as though the sputum would come up, but it does not.

Several members remarked here that Lachesis, Causticum, Sulphur, Arnica, and Stannum have the similar symptom of loose cough with no expectoration or scanty, there seeming to be a great deal of mucus in the chest, but it does not come up.

Dr. Scudder: A patient suffered from capillary bronchitis. Tart. em.[2] resulted in an eruption similar to small-pox in the genitals, and cured the case. In a case where there were five mucous valves all through the chest Tart. em. relieved.

Dr. Southwick: Have used Tart. em. successfully in loose coughs and in croup. In one case of a burn an eruption came out. Tart. em. helped the case and improved the appearance of the burn.

Dr. Seward: Used Tart. em. successfully in a trouble of the heart; throbbing, beating hard at times, and a sensation as if the heart would revolve.

Dr. E. P. Hussey, of Buffalo, N. Y., communicated to the Society through Dr. Hawley his desire that, for the purpose of advancing the interests of Homœopathy and its truths, this Society would publish a concise statement of the results of Neural Analysis. Whereupon Dr. E. P. Hussey was appointed a committee to prepare, with a view to its publication in pamphlet form for general distribution, and report to this Society at its December meeting, a paper containing a concise statement of the practical results of Neural Analysis, as applied to testing the effects of high potencies upon the human organism. Adjourned.

C. P. Jennings, *Secretary.*

