# Errata

**Published:** 1883-02

**Authors:** 


ERRATA.—In our December number (1882), p. 499, fifth line from the top, for "*five mucous valves*," read *fine mucous rales.*

In the same number some Lycopodium clinical cases were credited to Dr. C. W. Butler, when they should have been credited to Dr. J. S. Smith, of Westminster, Md.

